# Partisan and Geographic Variation in Emotional Responses to COVID-19 Vaccination on Social Media

**DOI:** 10.1001/jamanetworkopen.2026.15409

**Published:** 2026-06-01

**Authors:** Kokil Jaidka, Yuanyuan Wu, Anku Rani, Ozan Kuru, Reyhan Jamalova, Garrick Sherman, Sharath Chandra Guntuku

**Affiliations:** 1 National University of Singapore, Singapore; 2 Massachusetts Institute of Technology, Cambridge, Massachusetts; 3 University of Pennsylvania, Philadelphia; 4 National Institute on Drug Abuse, Bethesda, Maryland

## Abstract

**Importance:**

Public acceptance of the first COVID-19 vaccine administration was not uniform, yet the nature and county-level characteristics of heterogeneous emotional responses remain poorly characterized.

**Objective:**

To examine changes in social media emotional expression across US counties before and after the first COVID-19 vaccine administration, and whether changes varied by county-level partisanship and COVID-19 death rates.

**Design, Setting, and Participants:**

Cross-sectional study of geotagged COVID-19–related posts on the socia media platform Twitter from 3065 US counties from over 1.9 million unique users who created more than 18 million posts during the study period (September 1, 2020, to March 31, 2021), covering 100 days before and after first vaccine administration. Data were analyzed from January 2023 to April 2026.

**Exposure:**

Geotagged COVID-19–related social media posts during the 100-day periods before and after December 14, 2020.

**Main Outcomes and Measures:**

Levels of joy, sadness, anger, and fear expressed in posts, measured using the National Research Council of Canada Word-Emotion Association Lexicon.

**Results:**

After vaccine administration, fear decreased (β = −0.424; 95% CI, −0.510 to −0.338; *P* < .001; Cohen *d* = −0.20), joy increased (β = 0.683; 95% CI, 0.601 to 0.766; *P* < .001; Cohen *d* = 0.33), and anger increased (β = 0.445; 95% CI, 0.384 to 0.506; *P* < .001; Cohen *d* = 0.28). Sadness did not change significantly (β = −0.004; 95% CI, −0.075 to 0.067; *P* = .91). Democratic-leaning counties showed greater joy increases (β = 0.162; 95% CI, 0.115 to 0.209; *P* < .001), larger fear decreases (β = −0.066; 95% CI, −0.115 to −0.017; *P* = .007), and smaller anger increases (β = −0.042; 95% CI, −0.075 to −0.009; *P* = .009) than Republican-leaning counties. Higher county COVID-19 death rates were associated with greater fear decreases (β = −3.69 × 10^−6^; 95% CI, −6.03 × 10^−6^ to −1.35 × 10^−6^; *P* = .002); death rate did not significantly moderate joy or anger.

**Conclusions and Relevance:**

In this cross-sectional study of 18 million geotagged social media posts, the first COVID-19 vaccine administration was associated with increased joy and anger and decreased fear, with fear showing the greatest evidence of a vaccine-specific shift. These findings suggest that monitoring social media discourse can provide early signals of optimism, skepticism, and division, thereby informing targeted communication strategies.

## Key Points

**Question:**

How did collective emotional expressions on social media vary across US counties in response to the first COVID-19 vaccine administration?

**Findings:**

In this cross-sectional study of over 18 million geotagged social media posts from 3065 counties, joy and anger expressions increased, while fear decreased after the first vaccine dose on December 14, 2020. Democratic-leaning counties and those with higher COVID-19 death tolls showed larger increases in joy.

**Meaning:**

These findings suggest real-time social media monitoring can reveal heterogeneous emotional responses to public health milestones, informing targeted communication strategies.

## Teaser

This cross-sectional study evaluates changes in social media emotional expression across US counties before and after the first COVID-19 vaccine administration and whether changes varied by county-level partisanship and COVID-19 death rates.

## Introduction

Scientific breakthroughs have historically served as inflection points in public health, generating both relief and uncertainty in equal measure. The announcement of the Salk polio vaccine in 1955, greeted with public celebrations and church bells across the US, illustrates how a single medical milestone can trigger swift, emotionally charged collective responses. During the COVID-19 pandemic, the rapid development of COVID-19 vaccines, achieved in under 12 months compared with typical timelines of 10 to 15 years, represented an unprecedented scientific achievement, but also introduced uncertainties about safety and efficacy, which were observed in mass public opinion.^1^ While vaccines offered a pathway to normalcy, hesitancy and skepticism shaped a more complex reality. Existing literature has examined the development, distribution, and health outcomes of COVID-19 vaccines,^2,3,4^ yet it has paid less attention to the public’s immediate emotional reactions during the rollout. Understanding these responses is crucial, as collective emotions often shape both societal trust in science and the effectiveness of public health interventions. Prior research has shown that aggregate public responses to health crises show both relief and contention in the face of medical interventions,^5,6,7^ which suggests to us that collective emotional shifts observed on social media may serve as leading indicators of behavioral change.

This study aims to formally document and characterize public emotional responses to the initial administration of COVID-19 vaccines by analyzing expressions shared on social media platforms. Social media provides a unique lens on these dynamics: unlike traditional surveys, it captures spontaneous and large-scale expressions of emotion and opinion. Prior work has used social media data to examine emotional reactions during public health crises,^8,9,10,11^ but these studies often remain descriptive, leaving open questions about how emotions shift following major scientific events and how contextual factors influence these shifts. By focusing on emotions expressed in real time during vaccine rollout, this study provides evidence on how communities navigated one of the most consequential moments of the pandemic.

We focus on the vaccine rollout because it was a highly contested development, marked by divides across political, social, and health contexts. Research suggests that reactions to vaccines are shaped by structural factors such as political partisanship and local pandemic severity,^12,13,14,15^ yet whether these divides manifest in collective emotional expression at scale remains underexplored. To address this, we examine whether emotional shifts on social media following the first vaccine administration in the US varied systematically by county-level partisanship and COVID-19 death tolls. We focus on death toll rather than death rate because previous research indicates that individuals making important health decisions often prefer numeric information in the form of natural frequencies (absolute counts) over rates or probabilities.^16^ Presenting absolute numbers can reduce misinterpretation and may be more salient for public perception and emotional response.

To provide empirical evidence, this study uses an interrupted time series (ITS) analysis of 18 million geotagged social media posts to track changes in public expression of fear, anger, sadness, and joy, following the design of recent work documenting emotional dynamics during health crises.^16^ These 4 emotions, identified as key opposites in the Plutchik Wheel of Emotions, capture both negative and positive responses to uncertain events.^16,17^ Fear arises from perceived danger, anger from frustration attributed to others, sadness from loss, and joy from positive or hopeful developments. By analyzing shifts across these dimensions, we aim to provide new insights into how public emotions responded to the arrival of vaccines, thereby contributing to broader debates on public trust, polarization, and the societal reception of scientific breakthroughs.

## Methods

This cross-sectional study was approved by the National University of Singapore Department ethics review committee, and we followed the Strengthening the Reporting of Observational Studies in Epidemiology (STROBE) reporting guideline for cross-sectional studies. Informed consent was waived because the study used publicly available, deidentified social media data. This study had 3 objectives. First, after the first COVID-19 vaccine administration in the US, what were the differences in levels of fear, anger, sadness, and joy in COVID-19–related social media posts? Second, how did these changes in emotional expressions differ across counties with higher vs lower partisan support? Third, how did these changes in emotional expressions differ across counties with higher vs lower COVID-19 death counts? To answer these questions, we analyzed 18 million geotagged social media posts related to COVID-19, collected around the time of the vaccine rollout, and applied ITS analysis to estimate changes in emotional expression associated with the vaccine’s introduction. The study used publicly available data and was exempted from institutional review board approval.

### Data Collection and Preprocessing

The COVID-19-related social media posts analyzed in this study were derived from a larger dataset collected via the X (formerly Twitter, hereafter referred to as the platform) API over a 2-year period (March 2020 to March 2022). Data collection was guided by a systematic set of keywords (eg, *coronavirus*, *COVID-19*, and *nCoV2019*) and hashtags (eg, *#selfisolating*, *#StayTheFHome*, and *#CoronavirusPandemic*) (see eAppendix 1 in Supplement 1 for the complete list of search terms). For the present analysis, we focused on data spanning September 1, 2020, to March 31, 2021 (ie, approximately 100 days before and after the first COVID-19 vaccine administration in the US [December 14, 2020]). This temporal subset included more than 205 million social media posts (detailed counts available in eAppendix 2 and eTable 1 in Supplement 1). Social media posts were subsequently geolocated using location metadata provided by the platform API, resulting in a final dataset of over 18 million geotagged posts in the US (see eAppendix 3 in Supplement 1 for descriptive results). Before further analysis, we tokenized platform text using Social Tokenizer bundled with ekphrasis, a text processing pipeline designed for social networks.^18^ In addition, using ekphrasis, URLs, email addresses, percentages, currency amounts, phone numbers, usernames, emoticons, and times and dates contained in the platform, texts were normalized with metatokens such as *<url>*, *<email>*, *<user>*, and so on.

### Emotion Analysis

We analyzed posts’ emotional characteristics, which we automatically derived using the National Research Council of Canada (NRC) Word-Emotion Association Lexicon (EmoLex). The EmoLex contains English words associated with 8 basic emotions,^1,10^ with our research focusing on 4 emotions: sadness, joy, anger, and fear. We transformed each post into a vector by analyzing the post’s tokens against the token list, representing the percentage proportions of each feature in EmoLex. For the main findings, we offer a robustness check by including findings with the Linguistic Inquiry and Word Count lexicon.^1,11^ To validate our NRC-derived emotion scores, we independently classified a random sample of 28 075 social media posts using 2 transformer-based annotators: a pretrained DistilRoBERTa model fine-tuned for emotion classification^1,12^ and Llama 3.3-70B,^19^ accessed via the Groq API.^20^ Given the large number of posts with no emotion labels across the categories, reliability was assessed using a well-established probabilistic model-based framework^21^ that estimates annotator accuracy conditional on the latent true label (θ), alongside pairwise percentage agreement and Cohen κ. NRC reliability was satisfactory (θ ≥ 0.65) for the 4 primary emotions: fear (θ = 0.69), anger (θ = 0.71), joy (θ = 0.67), and sadness (θ = 0.70). This supports the validity of the lexicon-based approach for population-level emotion trend detection. Full validation statistics are reported in eAppendix 4 and eTable 2 in Supplement 1.

### Statistical Analysis

ITS analysis is gaining prominence for assessing the consequences of large-scale health interventions. It is often considered the optimal approach for establishing causality when randomized clinical trials (RCTs) are not feasible.^22^ Thus, we further conducted ITS regressions to quantify the associations between the major event (ie, the first COVID-19 vaccine was administered in the US) and the emotions shown in COVID-19 social media posts. We computed the mean score for each emotion found in posts grouped by day and county. The ITS models estimate piecewise linear trends (ie, 2 linear segments joined at the intervention date), testing for both an immediate level shift (β_2_) and a change in slope (β_3_). Figures display locally estimated scatterplot smoothing (LOESS) curves for visual presentation of nonparametric trends. Both approaches capture the same overall pattern: the LOESS curves provide a descriptive visualization while the ITS models provide formal hypothesis tests.

Altogether, the dataset includes 367 919 observation rows intended for ITS analysis. All emotion-related variables were scaled to values between 0 to 100. The independent variable was dichotomous, representing whether it was before or after the intervention (ie, December 14, 2020). To answer the first research question that investigates the emotional change before and after the main event, we built our first model:

*Y = β*_0_ + β_1_*T* + β_2_*X_t_* + β_3_*TX_t_* + (1|*county*).

In this model, T is the relative time distance from December 14, 2020. For instance, December 10, 2020 is denoted as −4 in the time variable, while December 18, 2020 is represented as 4. X is a dummy variable indicating whether the social media posts were published before (coded as 0) or after the intervention (coded as 1). The model also accounts for random effects due to county difference. The coefficients can be interpreted as follows: β_0_ represents the baseline or mean score before the intervention, β_1_ represents the change with each day progression, β_2_ stands for the intercept shift after the vaccine administration news intervention on emotion score, and β_3_ indicates the slope change after the intervention.

To answer the second research question, which aims to understand how emotional change differs at the county level due to partisanship or the COVID-19 death toll, we further obtained presidential precinct data for the 2020 general election from the New York Times and weekly US COVID-19 deaths by county from the US Centers for Disease Control and Prevention (CDC).^23,24^ The presidential precinct data include the number of votes received by Joseph Biden, the number of votes received by Donald Trump, and the total vote count. To calculate the vote margin at the county level, we first aggregated precinct-level data to the county level, and then we subtracted the votes received by Donald Trump from those received by Joseph Biden and divided the result by the total votes. This produces a vote margin ranging from −1 to 1, where higher values indicate stronger Democratic support. COVID-19 death toll was operationalized as cumulative deaths per 100 000 population as of December 9, 2020 (the most recent reporting date before vaccine administration), using county-level death data from the CDC and 2019 Census population estimates. The median (IQR) county death rate was 77.8 (39.4-129.6) per 100 000. Then, we built our second ITS model:

*Y = β*_0_ + β_1_*T* + β_2_*X_t_* + β_3_*TX_t_* + β_4_*Z* + β_5_*ZX_t_* + (1|*county*).

**Figure 1.**
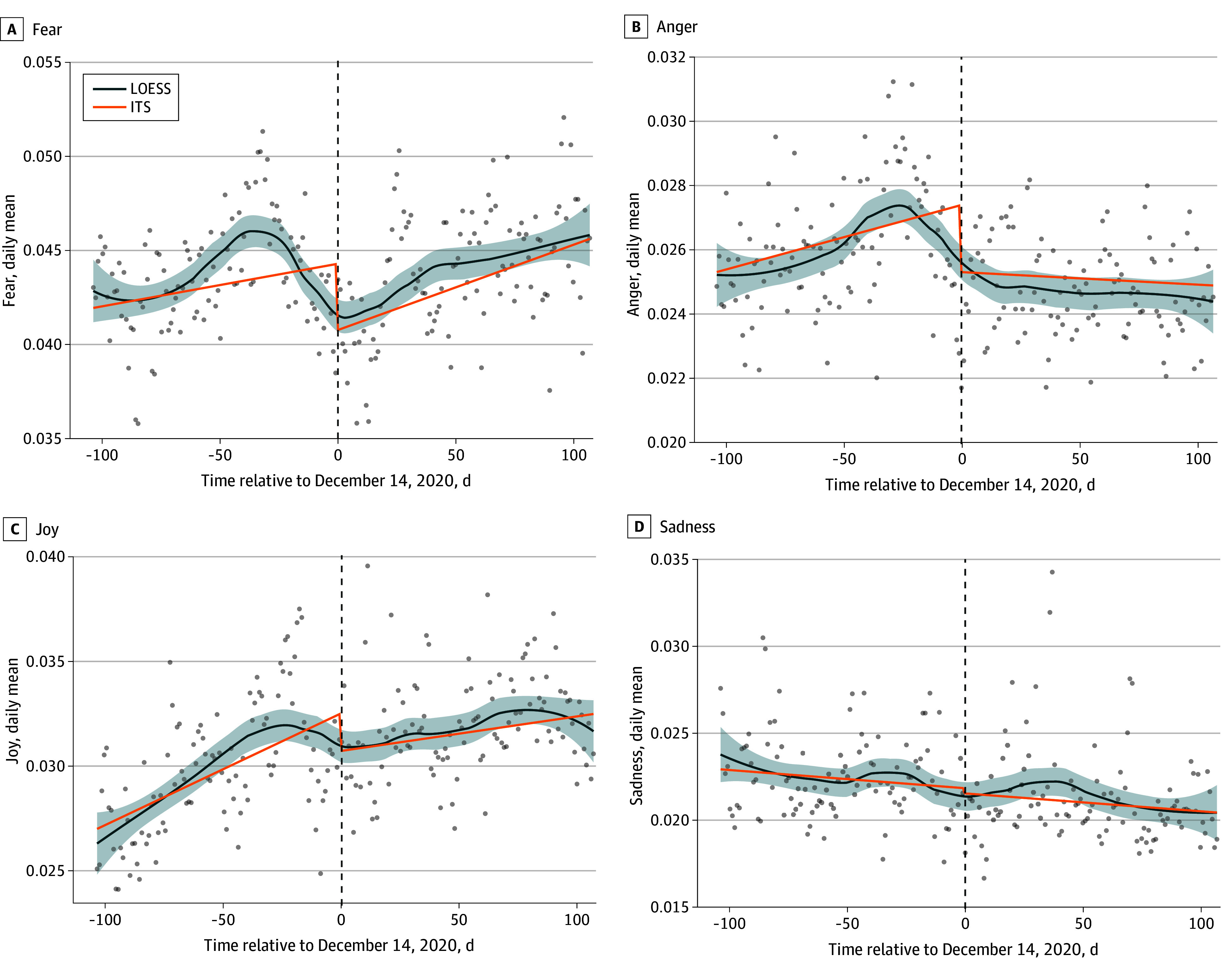
Line Graph Showing Correspondence Between Locally Estimated Scatterplot Smoothing (LOESS) and Interrupted Time Series (ITS) Piecewise Linear Model Fits for the 4 National Research Council of Canada Emotions Gray points indicate daily county-level means. Blue curve indicates LOESS (span = 0.5, 95% CI shaded). Orange line indicates ITS fixed-effect estimate: Y = β_0_ + β_1_ × time + β_2_ × intervention + β_3_ × (time × intervention). Dashed vertical line indicates vaccine rollout (December 14, 2020). LOESS and ITS agree closely on direction and timing of change in all 4 emotions, supporting the appropriateness of the piecewise linear specification.

Compared with the first model, we added Z, which represents either the vote margin or the COVID-19 death toll, and the interaction between Z and X. Other parts are the same as the first model. We focused on β_5_ of the interaction term between Z and X, as it demonstrates whether the change in emotion after the intervention varies depending on the vote margin or the COVID-19 death toll. Statistical analyses were conducted using R version 4.3.1 (R Project for Statistical Computing), with the lme4 package, version 1.1-35, for linear mixed-effects models and lmerTest (version 3.1-3) for obtaining *P* values via Satterthwaite degrees of freedom. Two-sided *P* values were used throughout, with statistical significance set at α = .05. To verify that the piecewise linear ITS assumption was not distorting observed patterns, we overlaid ITS model-estimated trajectories on LOESS-smoothed trends for each emotion; the 2 approaches agreed closely on the direction and timing of level and slope changes (Figure 1; eAppendix 5 and eFigure 1 in Supplement 1). All analyses were completed from January 2023 to April 2026.

## Results

### Sample Characteristics

The analytic sample included 18 million geotagged social media posts from 3062 counties over 213 days (eFigure 2 in Supplement 1). The median (IQR) daily post volume per county was 2.1 (1.3-7.1), with a mean (SD) of 28.6 (143.2), reflecting a highly right-skewed distribution associated with large urban counties (eTable 3 in Supplement 1). A total of 89.2% of counties had a mean of fewer than 30 posts per day. Social media post volume varied substantially by community type: big cities had a median of 642 posts per day compared with 1.1 for aging farmlands (eTable 4 in Supplement 1). The most common hashtags were *#covid19* (4.5 million uses), *#coronavirus* (715 000), and *#covid_19* (181 000). Notably, *#covidvaccine* usage increased 5.3-fold in the postvaccine period, while *#vote* and *#trumpknew* declined sharply, reflecting the shifting discourse from election to vaccination (eTable 5 in Supplement 1).

### Emotion Analysis

Among the 4 emotions analyzed, fear consistently had the highest percentage throughout the period (See eAppendix 6, eFigure 3, and eTable 6 in Supplement 1 for visualization and descriptive statistics). Fear showed an upward trend from October until mid-November 2020, followed by a decline until mid-December. At the beginning of September, there was little distinction between anger, joy, and sadness. However, joy gradually increased over time, while sadness showed a general decline, and anger remained relatively stable throughout the 7 months. Both joy and sadness reached a turning point around mid-December 2020.

### ITS Analysis

#### First COVID-19 Vaccine Administration Intervention Model

**Figure 2.**
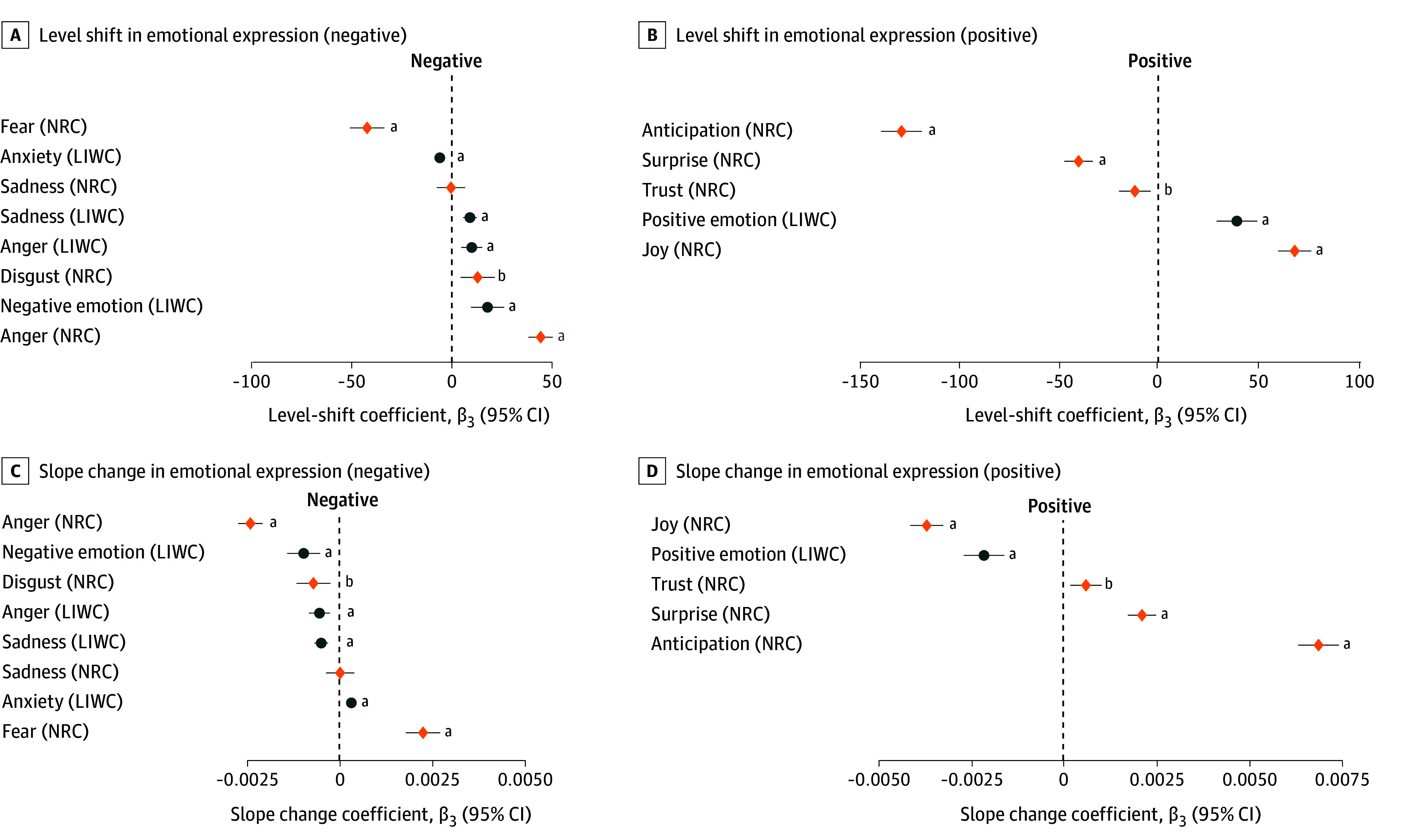
Dot Plots Showing Level-Shift and Slope-Change in Emotional Expression After the First COVID-19 Vaccine Administration Dot plots display interrupted time series estimates for 13 emotion measures from the National Research Council of Canada and LIWC lexicons. A, level-shift coefficients (β_2), indicating the immediate change after the first vaccine administration. B, slope-change coefficients (β_3), indicating the change in postintervention time trend. Points represent coefficient estimates, horizontal bars represent 95% CIs, blue circles indicate emotion measures from LIWC, and orange diamonds indicate emotion measures from the National Research Council of Canada. Emotions are separated into negative and positive valence panels, and the dashed vertical line indicates no effect. ^a^Indicates *P*<.001. ^b^Indicates *P*<.05.

The main effects are reported in Figure 2 (eAppendix 7 and eTables 7 and 8 in Supplement 1). As seen in panel A, after the first vaccine administration, fear expressions significantly decreased (*β_2_* = −0.424; 95% CI, −0.510 to −0.338; *P* < .001; approximate d = −0.196), whereas joy (*β_2_* = 0.683; 95% CI, 0.601 to 0.766; *P* < .001; approximate d = 0.325) and anger (*β_2_* = 0.445; 95% CI, 0.384 to 0.506; *P* < .001; approximate d = 0.284) increased. Sadness did not change significantly (*β_2_* = −0.004; 95% CI, −0.075 to 0.067; *P* = .91; approximate d = −0.002). These semistandardized effect ratios were small to modest in magnitude, with the largest effect observed for joy, followed by anger and fear.

Panel B shows that the main ITS model also showed significant time-by-intervention interactions for joy (*β_3_* = −0.004; 95% CI, −0.004 to −0.003; *P* < .001), fear (*β_3_* = 0.002; 95% CI, 0.002 to 0.003; *P* < .001), and anger (*β_3_* = −0.002; 95% CI, −0.003 to −0.002; *P* < .001). These estimates indicate that after the first COVID-19 vaccine administration, joy grew more slowly over time, fear increased more strongly over time, and anger declined more over time. The slope change for sadness was not statistically significant (*β_3_* ≈ 0.000; 95% CI, −0.000 to 0.000; *P* = .92).The increase in joy aligns with previous research indicating that scientific breakthroughs, such as vaccine rollouts, are often associated with relief and optimism in the public.^5^ The concurrent rise in anger supports findings from Carpiano et al,^6^ who documented polarized emotional reactions related to public health events, reflecting the contentious discourse and skepticism that have characterized the COVID-19 pandemic. The lack of a change in fear may be because sadness, as an emotion linked to long-term loss and grief, may be less responsive to abrupt positive developments, instead reflecting enduring consequences of the pandemic.^7^ In contrast, the observed decline in fear after the vaccine administration corroborates studies showing that increased perceived control and protective measures are associated with reductions in fear during health crises.^25,26,27^

#### Vote Margin

Furthermore, we examined how partisanship, as exhibited in vote margin, was associated with COVID-19 vaccine administration and emotions. We found significant interactions between vote margin and intervention for joy (β = 0.162; 95% CI, 0.115 to 0.209; *P* < .001), fear (β = −0.066; 95% CI, −0.115 to −0.017; *P* = .007), and anger (β = −0.042; 95% CI, −0.075 to −0.009; *P* = .009), but not for sadness (β = −0.032; 95% CI, −0.071 to 0.007; *P* = .14) (Table). This suggests that compared with counties that are Republican-leaning, Democrat-leaning counties in the US showed a larger increase in joy, a smaller increase in anger, and a larger decrease in fear after the first COVID-19 vaccine administration (Figure 2). This pattern reinforces earlier findings of partisan disparities in vaccine acceptance and attitudes toward public health measures and extends prior research by documenting that these divides are evident in real-time emotional expression on social media.^28,29,30,31^

**Table.** Summary of Interrupted Time Series (ITS) Models Estimating Emotion Scores

Model	Sadness		Joy		Fear		Anger	
	Estimate (SE)	*P* value	Estimate (SE)	*P* value	Estimate (SE)	*P* value	Estimate (SE)	*P* value
Main ITS model								
Constant	0.217 (0.007)	<.001	−0.959 (0.007)	<.001	−0.375 (0.011)	<.001	−0.347 (0.008)	<.001
Time	0	<.001	0	<.001	0	<.001	0	<.001
Intervention	−.004 (0.036)	.91	0.683 (0.042)	<.001	−.424 (0.044)	<.001	0.445 (0.031)	<.001
Intervention × time	0	.92	0	<.001	0	<.001	0	<.001
Cohen d	−0.002	NA	0.325	NA	−0.196	NA	0.284	NA
Partisanship-moderated ITS model								
Constant	0.221 (0.014)	<.001	−0.997 (0.016)	<.001	−0.385 (0.023)	<.001	−0.361 (0.018)	<.001
Time	0	<.001	0	<.001	0	<.001	0	<.001
Intervention	0.013 (0.019)	.16	0.76 (0.017)	<.001	−.403 (0.025)	<.001	0.469 (0.022)	<.001
Intervention × time	0	.50	0	<.001	0	<.001	0	<.001
Vote margin	0.001 (0)	.001	0.001 (0)	.10	0.001 (0.001)	.51	−.002 (0)	<.001
Vote margin × time	0 (0.02)	.13	0.162 (0.024)	<.001	−.066 (0.025)	.007	−.042 (0.017)	.009
Cohen d	.007	NA	.362	NA	-.186	NA	0.299	NA
Death toll–moderated ITS model								
Constant	0.218 (0.007)	<.001	−0.957 (0.007)	<.001	−0.377 (0.011)	<.001	−0.349 (0.009)	<.001
Intervention	0	<.001	0	<.001	0	<.001	0	<.001
Time	−.005 (0.015)	.75	0.68 (0.014)	<.001	−.420 (0.02)	<.001	0.446 (0.018)	.03
Death toll	0	.65	0	.31	0	.92	0	.17
Intervention × time	0 (0.001)	.76	0 (0.001)	<.001	0 (0.002)	<.001	0 (0.001)	.03
Intervention × death toll	0	.12	0	.10	0	.002	0	.48
Cohen d	−0.003	NA	0.324	NA	−0.194	NA	0.284	NA

Abbreviation: NA, not applicable.

#### Association With COVID-19 Death Toll

When COVID-19 death toll was operationalized as deaths per 100 000 population, higher county death rates were associated with significantly greater postvaccine declines in fear (interaction β = −3.69 × 10^−6^; 95% CI, −6.03 × 10^−^^6^ to −1.35 × 10^−^^6^; *P* = .002), trust (β = −3.55 × 10^−6^; 95% CI, 5.68 × 10⁻^6^ to −1.42 × 10⁻^6^; *P* = .001), and surprise (β = −3.57 × 10^−6^; 95% CI, −5.50 × 10⁻^6^ to −1.64 × 10⁻^6^; *P* < .001). Death rate did not significantly moderate the intervention effect on joy or anger . Results were broadly consistent when using log-transformed death rates, though only trust remained significant (eAppendix 8 and eTable 9 in Supplement 1). As shown in the Table, the ITS results reflected that compared with counties with low death tolls in the US, those with high death tolls witnessed a larger increase in joy but a smaller increase in anger. The findings are consistent with the health belief model and studies linking increased perceived severity to favorable attitudes toward vaccine uptake.^13,14,32^ Associations between post-vaccine emotion changes and subsequent county-level vaccination rates are reported in eAppendix 9, eTable 10, eTable 11, and eFigure 4 in Supplement 1.

## Discussion

This study makes 3 contributions to the literature on public responses to health interventions. First, whereas prior research has documented vaccine hesitancy and acceptance through surveys conducted weeks to months after vaccine availability,^6,7^ our analysis builds on top of real-time dashboards deployed by a team of researchers, including some of the authors, to study emotional shifts at the county level during the COVID-19 pandemic.^25^ Second, by finding an association between these shifts and county-level partisanship and pandemic severity, we demonstrate that a single national event produced systematically different emotional responses across communities. Third, our supplemental analyses clarify which of these shifts can be attributed specifically to the vaccine rollout vs broader temporal trends.

Of the 4 key emotions, fear provides the most compelling evidence of a vaccine-specific effect: placebo tests show its level-shift coefficient reversed sign from positive before December 11 to negative afterward (eAppendix 10 and eTable 12 in Supplement 1), a pattern corroborated by sensitivity analyses (eAppendix 11 and eTable 13 in Supplement 1) and significant moderation by county death rate. The partisan moderation of both emotions nonetheless indicates that these trajectories diverged systematically across the political spectrum.^28,29,30,31^ While these effect sizes are small to modest in absolute terms (Cohen *d* range: −0.196 to 0.325), we anticipate that when distributed across 18 million social media posts from nearly 2 million users, even a small mean shift in emotion scores represents a substantial change in the total volume of emotionally valenced content circulating in public discourse; this would be an effect that individual-level studies would not detect. Most importantly, the moderation analyses show that the same national event was associated with systematically different emotional trajectories across partisan and epidemiological contexts, with effect sizes for the interaction terms exceeding those of the main effects for joy in Democratic-leaning counties.

This study documents how emotions shifted in real time and varied across political and epidemiological contexts, building on earlier work linking polarization and perceived severity to vaccine attitudes^12,13,14,15^ and showing that such divides are also evident in collective emotional expression. More broadly, the findings contribute to scholarship on public trust in science by showing how breakthroughs can elicit both confidence and skepticism, underscoring the importance of monitoring emotions to anticipate how trust evolves during moments of uncertainty.

Our study design demonstrates the value of real-time emotion monitoring to guide health communication, complementing traditional surveys that cannot capture rapid changes.^33^ The finding that fear showed a vaccine-specific shift suggests that communication at the point of rollout may be most effective when directly addressing perceived threat rather than broadly boosting positive sentiment. The findings suggest that collective emotions are conditioned by local sociopolitical contexts; therefore, targeted, community-specific messaging may be more effective than uniform strategies.^34^

### Limitations

This study has several limitations that should be considered when interpreting the results. Naturally, our US-centric approach limits the generalizability of the findings. Although aggregation was necessary for integration with county-level indicators, we have shown that our results are consistent when frequency-based weights are incorporated into the models.

Second, while our analysis included code-mixed social media posts, the emotion lexicon used was designed for English-language text. Although over 96% of posts were in English, future work should consider emotion analysis tools tailored for multilingual data to enhance accuracy in more linguistically diverse contexts.

Third, only 1% to 2% of platform users enable geotagging, and these users tend to be younger, more urban, and more politically engaged than the general platform population.^27^ Sensitivity analyses weighting regressions by log daily post volume and excluding counties averaging fewer than 30 posts per day yielded consistent results (eAppendix 12 and eTables 14 and 15 in Supplement 1), suggesting that findings are not driven by low-volume counties.

Additionally, our study did not examine other contextual influences, such as misinformation, varying media coverage, or the role of influential public figures, which may have shaped emotional responses.^28^ Future research could explore these factors to build a more comprehensive understanding of how public discourse is formed around scientific breakthroughs.

## Conclusions

In this study, publicly available social media data were used to quantify collective emotional responses to the first COVID-19 vaccine administration in the US. Finding an association between emotional shifts and county-level political identity and local pandemic severity highlights how scientific breakthroughs provoke heterogeneous reactions rather than uniform consensus. Understanding these dynamics is critical, as public emotions are central to shaping trust in science and acceptance of health interventions. These findings suggest that monitoring social media discourse can provide early signals of optimism, skepticism, and division, thereby informing targeted communication strategies. As public health continues to depend on timely adoption of biomedical innovations, we encourage the use of similar real-time measures of collective sentiment for policy and communication planning, thereby ensuring effective public engagement with science.

## Notes

Supplement 1. eAppendix 1. Search Terms for COVID-19-Related Posts eAppendix 2. County Mapping Process eTable 1. Comparison of Post Counts Before and After Geographic County Mapping eAppendix 3. Overview of the Data eFigure 1. Number of Posts From September 1, 2020, to March 31, 2021 eTable 2. Overview of Post Volume, Users, and Counties eTable 3 Post Volume by 15 ACP Community Types eTable 4. Top 30 Hashtags With Pre-Post Ratio eAppendix 4. Descriptive Results of NRC Emotion Analysis eFigure 2. Daily Percentages of Emotional Expression in Posts eTable 5. Weekly and Overall Descriptive Statistics of NRC Emotions eAppendix 5. Validating NRC Lexicon-Based Emotion Labels eReferences eTable 6. Interannotator Agreement Statistics Across 6 Emotion Categories eAppendix 6. Model Specification Check: LOESS vs Piecewise Linear ITS eFigure 3. Correspondence Between LOESS Smoothing and ITS Piecewise Linear Model Fits for the 4 NRC Emotions eAppendix 7. Standardized Effect Sizes for All 13 Emotion Measures eTable 7. Standardized Effect Sizes for All 13 Emotions eTable 8. Cohen d eAppendix 8. Sensitivity Analyses With Shifted Intervention Dates eTable 9. ±7 and ±14 Day Intervention Shifts eAppendix 9. Robustness Checks: Post-Volume Weighting and County Exclusion eTable 10. Main Effects After Weighting by Post Volume eTable 11. Main Effects After Excluding Counties With Less Than 30 Posts eAppendix 10. Moderation by County-Level COVID-19 Death Rate eTable 12. Death Rate Per 100k Moderation, All 13 Emotions eAppendix 11. Correlation Between Emotions and Vaccination Rates eTable 13. Bivariate Correlation Between Change of Emotions and Vaccination Rates on March 12, 2021 eTable 14. Bivariate Correlation Between Change of Emotions and Vaccination Rates on April 30, 2021 eFigure 4. Bivariate Correlation Between Emotion Changes and Vaccination Rates eAppendix 12. Placebo Tests at Alternative Event Dates eTable 15. Placebo Tests With 8 Placebo Events eReferences.

Supplement 2. Data Sharing Statement
