# Supplementary material for: Partisan and Geographic Variation in Emotional Responses to COVID-19 Vaccination on Social Media
Source: JAMA Netw Open. 2026 Jun 1;9(6):e2615409. doi: 10.1001/jamanetworkopen.2026.15409 (PMC13227315; doi:10.1001/jamanetworkopen.2026.15409)
Supplement: Supplement 1. — eAppendix 1. Search Terms for COVID-19-Related Posts eAppendix 2. County Mapping Process eTable 1. Comparison of Post Counts Before and After Geographic County Mapping eAppendix 3. Overview of the Data eFigure 1. Number of Posts From September 1, 2020, to March 31, 2021 eTable 2. Overview of Post Volume, Users, and Counties eTable 3 Post Volume by 15 ACP Community Types eTable 4. Top 30 Hashtags With Pre-Post Ratio eAppendix 4. Descriptive Results of NRC Emotion Analysis eFigure 2. Daily Percentages of Emotional Expression in Posts eTable 5. Weekly and Overall Descriptive Statistics of NRC Emotions eAppendix 5. Validating NRC Lexicon-Based Emotion Labels eReferences eTable 6. Interannotator Agreement Statistics Across 6 Emotion Categories eAppendix 6. Model Specification Check: LOESS vs Piecewise Linear ITS eFigure 3. Correspondence Between LOESS Smoothing and ITS Piecewise Linear Model Fits for the 4 NRC Emotions eAppendix 7. Standardized Effect Sizes for All 13 Emotion Measures eTable 7. Standardized Effect Sizes for All 13 Emotions eTable 8. Cohen d eAppendix 8. Sensitivity Analyses With Shifted Intervention Dates eTable 9. ±7 and ±14 Day Intervention Shifts eAppendix 9. Robustness Checks: Post-Volume Weighting and County Exclusion eTable 10. Main Effects After Weighting by Post Volume eTable 11. Main Effects After Excluding Counties With Less Than 30 Posts eAppendix 10. Moderation by County-Level COVID-19 Death Rate eTable 12. Death Rate Per 100k Moderation, All 13 Emotions eAppendix 11. Correlation Between Emotions and Vaccination Rates eTable 13. Bivariate Correlation Between Change of Emotions and Vaccination Rates on March 12, 2021 eTable 14. Bivariate Correlation Between Change of Emotions and Vaccination Rates on April 30, 2021 eFigure 4. Bivariate Correlation Between Emotion Changes and Vaccination Rates eAppendix 12. Placebo Tests at Alternative Event Dates eTable 15. Placebo Tests With 8 Placebo Events eReferences. [file jamanetwopen-e2615409-s001.pdf]

Supplemental Online Content

Jaidka K, Wu Y, Rani A. Partisan and geographic variation in emotional responses to the COVID-19 vaccination on social media. *JAMA Netw Open*. 2026;9(5):e2615409. doi:10.1001/jamanetworkopen.2026.15409

eAppendix 1. Search Terms for COVID-19-Related Posts
eAppendix 2. County Mapping Process
eTable 1. Comparison of Post Counts Before and After Geographic County Mapping
eAppendix 3. Overview of the Data
eAppendix 4. Validating NRC Lexicon-Based Emotion Labels
eReferences
eTable 2. Interannotator Agreement Statistics Across 6 Emotion Categories
eAppendix 5. Model Specification Check: LOESS vs Piecewise Linear ITS
eFigure 1. Correspondence Between LOESS Smoothing and ITS Piecewise Linear Model Fits for the 4 NRC Emotions
eFigure 2. Number of Posts From September 1, 2020, to March 31, 2021
eTable 3. Overview of Post Volume, Users, and Counties
eTable 4 Post Volume by 15 ACP Community Types
eTable 5. Top 30 Hashtags With Pre-Post Ratio
eAppendix 6. Descriptive Results of NRC Emotion Analysis
eFigure 3. Daily Percentages of Emotional Expression in Posts
eTable 6. Weekly and Overall Descriptive Statistics of NRC Emotions
eAppendix 7. Standardized Effect Sizes for All 13 Emotion Measures
eTable 7. Standardized Effect Sizes for All 13 Emotions
eTable 8. Cohen d
eAppendix 8. Moderation by County-Level COVID-19 Death Rate
eTable 9. Death Rate Per 100k Moderation, All 13 Emotions
eAppendix 9. Correlation Between Emotions and Vaccination Rates
eTable 10. Bivariate Correlation Between Change of Emotions and Vaccination Rates on March 12, 2021
eTable 11. Bivariate Correlation Between Change of Emotions and Vaccination Rates on April 30, 2021
eFigure 4. Bivariate Correlation Between Emotion Changes and Vaccination Rates
eAppendix 10. Placebo Tests at Alternative Event Dates
eTable 12. Placebo Tests With 8 Placebo Events
eAppendix 11. Sensitivity Analyses With Shifted Intervention Dates
eTable 13. ±7 and ±14 Day Intervention Shifts
eAppendix 12. Robustness Checks: Post-Volume Weighting and County Exclusion
eTable 14. Main Effects After Weighting by Post Volume
eTable 15. Main Effects After Excluding Counties With Less Than 30 Posts
eReferences

This supplemental material has been provided by the authors to give readers additional information about their work.

© 2026 Jaidka K et al. *JAMA Network Open.*

eAppendix 1. Search Terms for COVID-19-Related Posts

The following keywords and hashtags were used for searching COVID-19-related posts on X (Twitter): coronavirus, coronavirus, covid-19, nCoV2019, #coronavirus, #coronovirus, #coronavirusoutbreak, #covid19us, #2019-nCoV, #Novel, #wuhan, #Chinese, #China, #Outbreak, #Medical, #Mask, #donate, #border, #Covid_19, #CoronavirusPandemic, #COVID19, #CoronaVirusUpdate, #COVD19, #Corona, #CoronaOutbreak, #CODVID19, #SocialDistancing, #selfisolating, #StayTheFHome, #coronapocolypse

eAppendix 2. County Mapping Process

Tweets were geolocated using X (formerly Twitter)'s API through a Hadoop-based county mapping process. This geolocation procedure substantially reduced the dataset size, as demonstrated in eTable 1 below.

eTable 1. Comparison of Post Counts Before and After Geographic County Mapping

Year -Month	Tweet Counts before county-mapping	Tweet Counts after county-mapping
2020.09	34,605,250	2,752,139
2020.10	40,695,440	4,179,498
2020.11	26,722,580	2,658,463
2020.12	29,661,980	3,011,351
2021.01	30,865,957	2,262,549
2021.02	19,655,226	1,529,620
2021.03	23,663,426	1,721,310
Overall	205,869,859	18,114,930

eAppendix 3. Overview of the Data

Our analysis included more than 18 million tweets from nearly 2 million users. eFigure 1 shows a fluctuating trend in tweet volume from September 2020 to March 2021. The volume peaked in October 2020, followed by a slight decline in November. December 2020 saw another peak with more than 3 million tweets, but the trend reversed with lower volumes from January to March 2021. This trend is also depicted in Figure 1, where early October 2020 shows a sharp spike, likely linked to news surrounding then-President Donald Trump and First Lady Melania Trump testing positive for COVID-19. Tweet volume dropped significantly in October and remained relatively stable, ranging between 50 thousand to 100 thousand tweets per day until January 2021. Afterward, tweet counts steadily declined to around 50 thousand daily by February and March 2021.

As shown in eTable 2, in line with the tweet volume trend, the number of unique users each month also exhibits fluctuations. The highest count, exceeding 800 thousand users, occurs in October 2020, while the lowest, with 420 thousand users, is observed in February 2021. Regarding the geolocation of these posts, the tweets originate from 3,065 unique counties in the U.S., averaging approximately 2,800 counties each month. eTable 3 reports the tweet volume by community type, and eTable 4 reports the top 30 hashtags with their pre/post ratios before and after the intervention on December 14, 2020.

eAppendix 4. Validating NRC Lexicon-Based Emotion Labels

To assess the reliability of the NRC lexicon-based emotion labels used in the primary analysis, we classified a random, date-stratified sample of 29,057 geotagged tweets using two transformer-based emotion classifiers: (1) a pretrained DistilRoBERTa model fine-tuned for emotion classification (Hartmann, 2022), which outputs continuous probability scores across 7 emotion categories; and (2) Llama 3.3-70B accessed via the Groq API, which was prompted to assign probability scores across the same categories. Both transformer classifiers were applied at the tweet level and aggregated to county-day means to match the unit of analysis in the primary ITS models.

Emotion presence was operationalised as a binary label per emotion per tweet (NRC word count > 0; transformer probability > 0.10). Unlike chance-corrected metrics such as Cohen's kappa, which have wide error bounds and are sensitive to label prevalence, model-based inference estimates each annotator's accuracy conditional on the latent true label — reported here as θ, the average of the true-positive rate P(present | truly present) and true-negative rate P(absent | truly absent) for each annotator (Passonneau and Carpender, 2014). θ has been widely applied to validate label annotations in computational linguistics (Cheng et al., 2024; Davani et al., 2022; Jaidka, 2022; Jaidka et al., 2024, Verma et al., 2025).

Pairwise percentage agreement and Cohen's kappa across the three annotators are reported in eTable 2. Three-way Fleiss' κ = 0.158 (P < .001); pairwise kappas ranged from κ = 0.131 (NRC vs. Llama 3.3-70B) to κ = 0.242 (RoBERTa vs. Llama 3.3-70B). These values reflect the well-documented tendency for emotion annotation systems to show modest agreement on short social media texts, particularly when methods differ in whether they capture explicit emotional vocabulary (lexicon-based) versus contextual sentiment (transformer-based).

Following Passonneau and Carpenter's (2014) recommended threshold of θ ≥ 0.65 for acceptable annotation quality, NRC label reliability was acceptable for the four emotions central to the primary ITS analysis. For fear — the emotion showing the most theoretically meaningful and replication-consistent shift in the main findings — NRC achieved θ = 0.69 (P(present|present) = 0.708; P(absent|absent) = 0.662). For anger, NRC θ = 0.71 (0.577; 0.836). For joy, θ = 0.67 (0.564; 0.783). For disgust, θ = 0.70 (0.512; 0.889). Sadness fell below the threshold (θ = 0.62), consistent with its null result in the primary analysis and prior evidence that sadness is less reliably captured by lexicon-based methods in COVID-19 social media data. Surprise showed poor NRC reliability (θ = 0.47), driven by a low true-positive rate (P(present|present) = 0.221), indicating that the NRC lexicon frequently misses surprise-laden tweets — a known limitation of word-count approaches for this emotion category.

Pairwise percentage agreement between NRC and the transformer annotators exceeded 70% for anger (NRC/RoBERTa = 71.7%; NRC/Llama 3.3-70B = 66.7%) and disgust (77.1%; 70.1%), and approached 70% for joy (73.1%; 69.4%). Agreement was lower for fear (64.0%; 55.8%), sadness (63.5%; 62.6%), and surprise (59.3%; 67.5%), reflecting both the base rate differences across annotators and the fundamentally different constructs being measured. Taken together, the θ values and pairwise agreement patterns support the validity of NRC labels for anger, fear, joy, and disgust in the context of population-level trend detection.

The divergence between NRC and the transformer classifiers is consistent with a known methodological distinction: NRC detects explicit emotion words (e.g., “afraid,” “happy”), while transformer models infer contextual sentiment from surrounding language. After vaccine rollout, users may have reduced explicit fear vocabulary (NRC detects a decrease) while the overall contextual tone of tweets about COVID-19 remained fearful or anxious (transformers detect an increase). Additionally, the sampled tweets are disproportionately from high-volume urban counties, which may not reflect the emotional trajectory of the full 3,062-county panel on which the primary results are based.

eTable 2. Interannotator Agreement Statistics Across 6 Emotion Categories

Emotion	NRC % present	RoBERTa % present	Llama % present	NRC/Roberta % agree	NRC/Llama % agree	Roberta/ Llama % agree	NRC θ	RoBERTa θ	Llama θ
Anger	28.8	21.5	41.0	71.7	66.7	72.4	0.71	0.78	0.87
Disgust	20.6	15.7	32.1	77.1	70.1	73.7	0.70	0.74	0.83
Fear	42.4	19.8	37.0	64.0	55.8	65.3	0.69	0.85	0.65
Joy	26.4	12.9	20.3	73.1	69.4	82.9	0.67	0.85	0.84

© 2026 Jaidka K et al. *JAMA Network Open*.

Emotion	NRC % present	RoBERTa % present	Llama % present	NRC/Roberta % agree	NRC/Llama % agree	Roberta/ Llama % agree	NRC θ	RoBERTa θ	Llama θ
Sadness	35.6	23.3	22.3	63.5	62.6	77.0	0.62	0.81	0.74
Surprise	27.8	26.1	9.6	59.3	67.5	73.0	0.47	0.76	0

Notes: θ = average of P(present | truly present) and P(absent | truly absent) from Passoneau–Litman EM model. Threshold for acceptable reliability: θ ≥ 0.65 (Passoneau & Litman, 2014). Cohen's κ: NRC vs. RoBERTa = 0.151; NRC vs. Llama = 0.131

© 2026 Jaidka K et al. *JAMA Network Open*.

eAppendix 5. Model Specification Check: LOESS vs Piecewise Linear ITS

The main analyses is based on piecewise linear interrupted time-series (ITS) models. However, LOESS and ITS operate on different assumptions, that is, non-parametric local smoothing versus global piecewise linearity, which can lead to apparent discrepancies between the two, especially with regards to whether the linear model misrepresents the underlying trend.

To address this, eFigure 1 overlays ITS fixed-effect predictions on LOESS-smoothed trajectories for all four NRC emotions (fear, anger, joy, sadness). Fixed-effect predictions were computed from model coefficients applied to daily county-level means, excluding random effects, to represent the population-average trend.

The two approaches agree closely on the direction, magnitude, and timing of change for all four emotions. The ITS line captures the level shift at rollout with high fidelity to the LOESS curve, and the post-rollout slope correctly tracks the LOESS trajectory through day +100. The main discrepancy, as expected, is that ITS does not reproduce the non-linear pre-intervention oscillations (most visibly the ~day −30 peak in fear and anger), which reflect real-world events during the pre-period (e.g., the U.S. election, Thanksgiving) rather than the intervention effect of interest. These pre-period fluctuations are absorbed into the intercept and pre-intervention slope estimate and do not bias the β_2 or β_3 estimates, which are identified entirely from the discontinuity at day 0. Together, the visual correspondence supports the appropriateness of the piecewise linear specification for inference.

eFigure 1. Correspondence Between LOESS Smoothing and ITS Piecewise Linear Model Fits for the 4 NRC Emotions

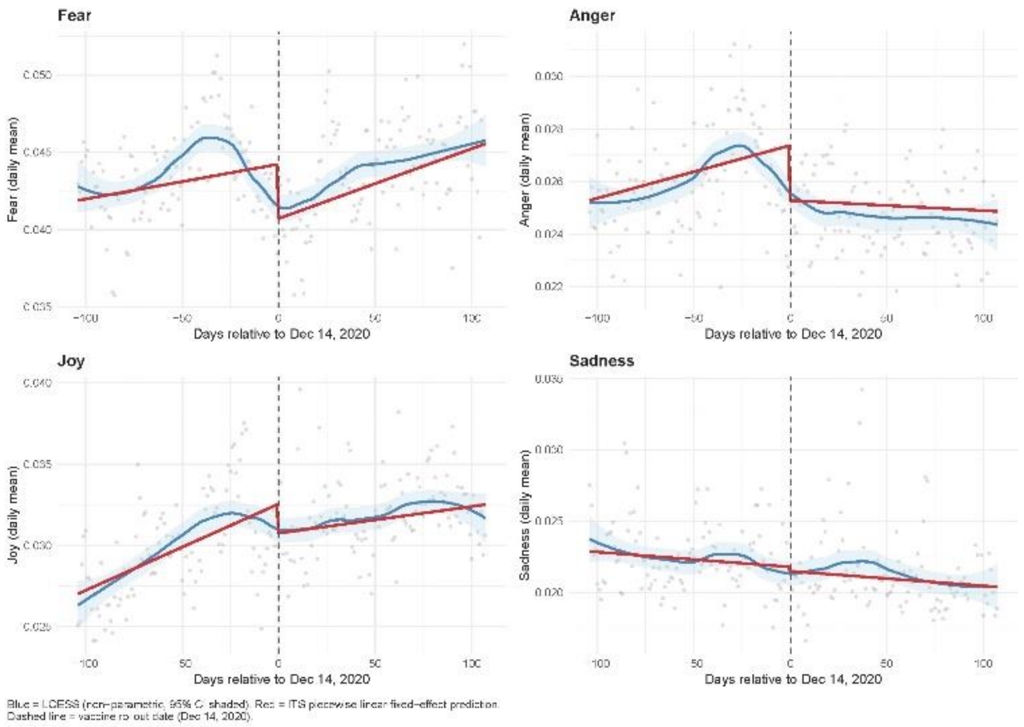

Note: Gray points = daily county-level means. Blue curve = LOESS (span = 0.5, 95% CI shaded). Red line = ITS fixed-effect prediction: Y = β_0 + β_1·time + β_2·intervention + β_3·(time × intervention). Dashed vertical line = vaccine rollout (December 14, 2020). LOESS and ITS agree closely on direction and timing of change in all four emotions, supporting the appropriateness of the piecewise linear specification.

© 2026 Jaidka K et al. *JAMA Network Open*.

eFigure 2. Number of Posts From September 1, 2020, to March 31, 2021

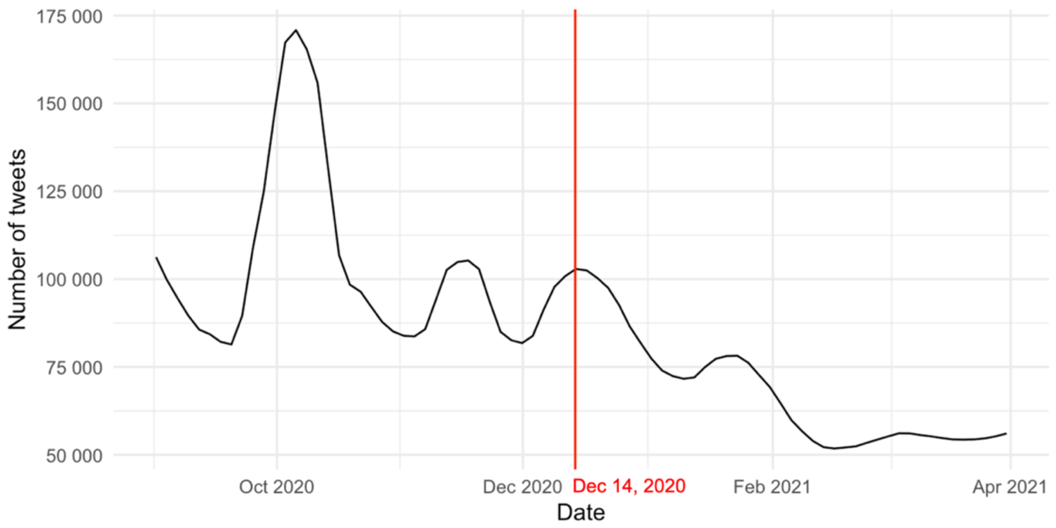

eTable 3. Overview of Post Volume, Users, and Counties

Month - Year	Tweet Counts	Users	Counties
Sep-2020	2,752,139	666,412	2,894
Oct-2020	4,179,498	816,645	2,906
Nov-2020	2,658,463	683,049	2,878
Dec-2020	3,011,351	699,577	2,887
Jan-2021	2,262,549	592,629	2,810
Feb-2021	1,529,620	420,726	2,694
Mar-2021	1,721,310	468,549	2,718
Total	18,114,930	1,948,690	3,065

Note. “Tweet counts” represent the total number of tweets posted each month, while “users” and “counties” refer to the number of unique users and unique counties, respectively, that contributed tweets.

eTable 4. Post Volume by 15 ACP Community Types

2023 Typology	Median tweets per day	Mean tweets per day	SD tweets per day	Number of counties	% Counties < 30 tweets
Big Cities	642.5	964.2	1053.4	48	0
Urban Burbs	73.4	120.7	136.7	112	20.5
College Towns	22.6	40.7	49.7	171	60.2
Middle Suburbs	17.9	25.1	18.4	57	70.2
Exurbs	8.9	22.1	33	196	78.1
Military Posts	8.1	18.3	27.7	77	88.3
Rural Middle America	2.1	4	6.2	628	99
Graying America	2	7.2	20.7	384	95.3
Working Class Country	1.9	3.5	5.6	276	98.9
Hispanic Centers	1.9	17.1	59.4	171	90.6
LDS Enclaves	1.9	15.8	62.2	37	94.6
African American South	1.5	9.8	42.3	263	95.4

© 2026 Jaidka K et al. *JAMA Network Open.*

Evangelical Hubs	1.5	2.3	2.9	371	99.7
Native American Lands	1.4	2.7	2.9	38	100
Aging Farmlands	1.1	1.3	0.5	233	100

eTable 5. Top 30 Hashtags With Pre-Post Ratio

Hashtag	Pre_vaccine	Post_vaccine	Total	Post:Pre Ratio
#covid19	2692808	1815003	4507811	0.67
#coronavirus	493984	221495	715479	0.45
#covid_19	103502	77849	181351	0.75
#wearamask	87317	37683	125000	0.43
#covid	69916	49017	118933	0.7
#1	103318	5504	108822	0.05
#vaccine	36675	70699	107374	1.93
#pandemic	55921	40288	96209	0.72
#trump	62186	15247	77433	0.25
#covidiots	50465	18728	69193	0.37
#breaking	32546	24635	57181	0.76
#socialdistancing	36269	16536	52805	0.46
#covidvaccine	8121	42727	50848	5.26
#vote	47001	948	47949	0.02
#maskup	30620	16787	47407	0.55
#trumpvirus	34664	5209	39873	0.15
#sarscov2	20209	19359	39568	0.96
#trumpknew	37165	401	37566	0.01
#healthcareheroes	14742	20534	35276	1.39
#health	17546	15635	33181	0.89
#corona	17602	15030	32632	0.85
#maga	27005	4725	31730	0.17
#healthcare	18943	12574	31517	0.66
#vaccines	9670	20724	30394	2.14
#masks	21273	5545	26818	0.26
#florida	11746	11238	22984	0.96
#stayhome	13142	9308	22450	0.71
#scotus	18161	4248	22409	0.23
#bidenharris2020	21542	762	22304	0.04
#biden	11965	10071	22036	0.84

eAppendix 6. Descriptive Results of NRC Emotion Analysis

We present the descriptive results of the NRC emotion analysis in the eFigure 3 and eTable 6 below, including the weekly median, mean, and standard deviation for each emotion (anger, fear, joy, and sadness), as well as the overall scores (median, mean, and standard deviation) for each emotion across the entire period.

eFigure 3. Daily Percentages of Emotional Expression in Posts

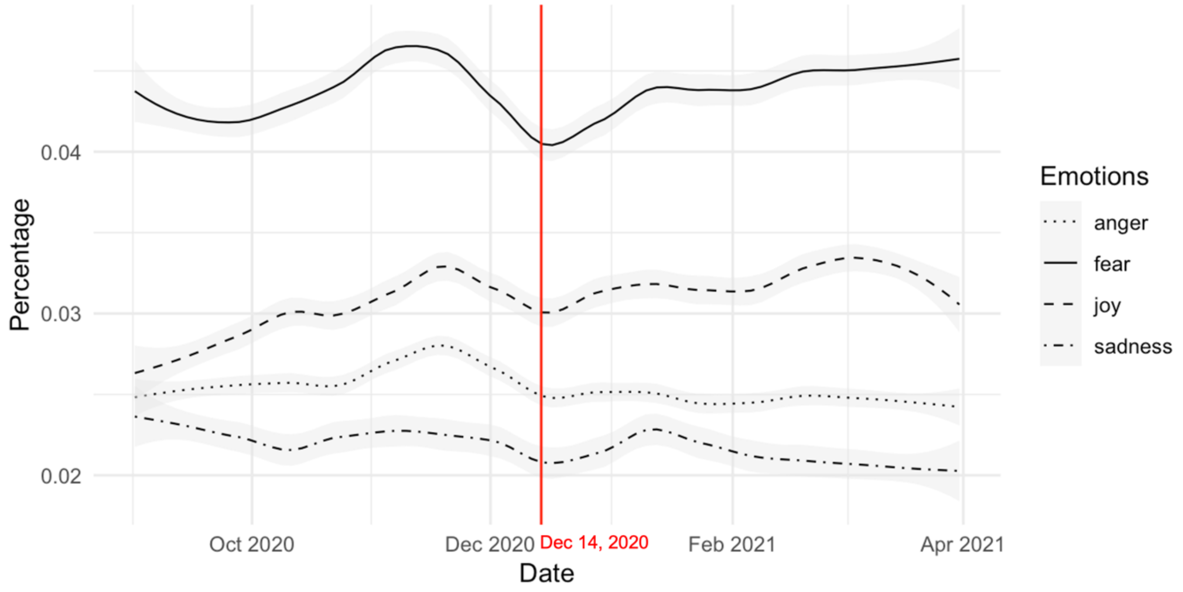

eTable 6. Weekly and Overall Descriptive Statistics of NRC Emotions

		Anger			Fear			Joy			Sadness		
Week	Start Date	*Med*	*M*	*SD*	*Med*	*M*	*SD*	*Med*	*M*	*SD*	*Med*	*M*	*SD*
1	01/09/2020	.024	.025	.016	.042	.044	.023	.026	.028	.019	.021	.023	.016
2	08/09/2020	.024	.025	.014	.042	.043	.020	.024	.026	.017	.022	.023	.015
3	15/09/2020	.024	.025	.014	.039	.039	.021	.025	.026	.019	.022	.024	.017
4	22/09/2020	.025	.027	.015	.041	.043	.023	.026	.027	.020	.023	.024	.019
5	29/09/2020	.025	.026	.014	.041	.042	.018	.030	.031	.018	.020	.021	.015
6	06/10/2020	.025	.026	.013	.042	.043	.018	.029	.030	.017	.020	.021	.014
7	13/10/2020	.023	.024	.015	.042	.043	.019	.029	.031	.019	.020	.021	.017
8	20/10/2020	.025	.026	.018	.042	.043	.019	.028	.030	.022	.022	.023	.015
9	27/10/2020	.025	.026	.015	.044	.045	.023	.027	.029	.021	.023	.024	.026
10	03/11/2020	.024	.026	.019	.045	.046	.024	.030	.032	.023	.020	.022	.024
11	10/11/2020	.028	.029	.016	.048	.049	.023	.030	.031	.020	.020	.021	.022
12	17/11/2020	.028	.029	.019	.044	.045	.025	.033	.035	.030	.021	.022	.018
13	24/11/2020	.027	.028	.016	.044	.045	.023	.032	.034	.024	.023	.025	.019
14	01/12/2020	.025	.026	.015	.041	.042	.021	.027	.028	.020	.019	.021	.023
15	08/12/2020	.023	.024	.016	.041	.042	.022	.029	.031	.024	.020	.021	.019
16	15/12/2020	.024	.025	.015	.040	.041	.024	.028	.030	.022	.019	.020	.015
17	22/12/2020	.024	.025	.015	.037	.039	.020	.028	.031	.026	.018	.020	.018
18	29/12/2020	.025	.026	.017	.040	.041	.022	.029	.031	.020	.022	.023	.019

© 2026 Jaidka K et al. *JAMA Network Open*.

19	05/01/2021	.024	.025	.019	.045	.047	.026	.030	.032	.024	.020	.022	.018
20	12/01/2021	.025	.026	.014	.043	.045	.025	.030	.032	.022	.020	.022	.022
21	19/01/2021	.023	.024	.014	.040	.042	.021	.029	.031	.020	.021	.024	.020
22	26/01/2021	.023	.024	.017	.042	.043	.022	.027	.030	.021	.020	.021	.016
23	02/02/2021	.023	.024	.015	.044	.045	.022	.031	.033	.021	.019	.021	.021
24	09/02/2021	.024	.026	.015	.043	.044	.023	.030	.032	.024	.018	.020	.015
25	16/02/2021	.023	.025	.018	.044	.046	.025	.030	.032	.023	.020	.023	.021
26	23/02/2021	.023	.024	.014	.043	.045	.024	.031	.033	.022	.018	.020	.019
27	02/03/2021	.024	.026	.019	.043	.045	.026	.032	.034	.022	.018	.019	.015
28	09/03/2021	.023	.024	.015	.041	.043	.024	.032	.034	.021	.019	.021	.017
29	16/03/2021	.023	.025	.017	.045	.047	.026	.030	.033	.023	.019	.021	.016
30	23/03/2021	.023	.024	.015	.044	.046	.025	.029	.031	.023	.018	.021	.017
31	30/03/2021	.024	.024	.013	.043	.046	.023	.028	.030	.019	.018	.019	.013
	Overall	.024	.025	.016	.042	.044	.023	.029	.031	.022	.020	.022	.018

Note. *Med* = Median; *M* = Mean; *SD* = Standard deviation. The final row reports overall emotion scores aggregated across the full time period (September 2020 to March 2021)

© 2026 Jaidka K et al. *JAMA Network Open*.

eAppendix 7. Standardized Effect Sizes for All 13 Emotion Measures

eTables 7 and 8 report pre-intervention means, standard deviations, and post-intervention means for all 13 emotion measures (8 NRC and 5 LIWC) on the 0–100 scale. For each emotion, we report the estimated level-shift coefficient (β_2) with its standard error, 95% confidence interval, and P value, as well as a semi-standardized effect ratio computed as β_2 divided by the pre-intervention standard deviation and partial η^2. We also report the estimated slope-change coefficient (β_3) with its standard error, P value, and partial η^2.

eTable 7. Standardized Effect Sizes for All 13 Emotions: β, 95% CI

Emotion	Pre Mean	Pre SD	Post Mean	Beta level shift	SE level	95% CI low	95% CI high	P level
**NRC lexicon**								
Fear	4.36	2.16	4.37	-0.424	0.044	-0.510	-0.338	< .001
Anger	2.61	1.57	2.47	0.445	0.031	0.384	0.506	< .001
Joy	2.99	2.10	3.18	0.683	0.042	0.601	0.766	< .001
Sadness	2.25	1.88	2.11	-0.004	0.036	-0.075	0.067	0.91
**LIWC lexicon**								
Positive emotion	2.03	2.74	1.94	0.394	0.052	0.292	0.496	< .001
Sadness	0.32	0.94	0.25	0.091	0.017	0.057	0.125	< .001
Negative emotion	1.60	2.17	1.31	0.179	0.042	0.096	0.262	< .001
Anger	0.53	1.34	0.42	0.100	0.027	0.047	0.153	< .001
Anxiety	0.27	0.69	0.23	-0.060	0.014	-0.086	-0.033	< .001

eTable 8. Cohen d, partial η²

Emotion	Cohen d level	Partial eta2 level	Beta slope change	SE slope	P slope	Partial eta2 slope
**NRC lexicon**						
**Fear**	-0.196	0.000248	0.002259	0.000237	< .001	0.000243
**Anger**	0.284	0.000549	-0.002404	0.000167	< .001	0.000554
**Joy**	0.325	0.000700	-0.003682	0.000227	< .001	0.000704
**Sadness**	-0.002	0.000000	0.000019	0.000195	0.92	0.000000
**LIWC lexicon**						
**Positive emotion**	0.144	0.000154	-0.002137	0.000279	< .001	0.000157
**Sadness**	0.097	0.000073	-0.000491	0.000094	< .001	0.000074
**Negative emotion**	0.082	0.000048	-0.000965	0.000227	< .001	0.000048
**Anger**	0.075	0.000037	-0.000537	0.000145	< .001	0.000037
**Anxiety**	-0.087	0.000053	0.000323	0.000073	< .001	0.000053

Notes. Cohen's d computed as β_2 / pre-intervention SD. Partial η^2 approximated as $t^2 / (t^2 + df)$.

© 2026 Jaidka K et al. *JAMA Network Open.*

eAppendix 8. Moderation by County-Level COVID-19 Death Rate

eTable 9 reports results from models including county-level cumulative COVID-19 deaths per 100,000 population as a moderator of the intervention effect. For each of the 13 emotions, we report the main intervention coefficient and the intervention × death rate interaction term with P values. Main intervention effects (β_2) were essentially unchanged from the primary models after including the death rate covariate. The intervention × death rate interaction was significant ($p < .05$) for 3 of 13 emotions in the per-100k specification. Counties with higher COVID-19 death rates showed larger post-vaccine decreases in fear, trust, and surprise, and smaller increases in positive emotion. In the log-transformed specification, only trust reached significance ($P < .001$). Joy and anger moderation by death rate were not significant in either specification ($P = .12$ and $P = .53$, respectively), indicating that the post-vaccine shifts in those emotions were consistent across counties regardless of local death toll severity.

eTable 9. Death Rate Per 100k Moderation, All 13 Emotions

Emotion	β Interv	β Interact	β Death Rate	β Interv × DR	p Interv	p Interact	p Death Rate	p Interv × DR
Fear	-0.420	2.240e-05	-1.320e-07	-3.690e-06	< .001	< .001	0.938	0.002
Anger	0.446	-2.410e-05	1.700e-06	-5.280e-07	< .001	< .001	0.109	0.529
Joy	0.680	-3.670e-05	1.960e-06	1.750e-06	< .001	< .001	0.235	0.124
Sadness	-0.005	2.610e-07	-3.440e-07	1.550e-06	0.884	0.893	0.758	0.114

© 2026 Jaidka K et al. *JAMA Network Open*.

eAppendix 9. Correlation Between Emotions and Vaccination Rates

While not the primary focus of this study, we examined bivariate correlations between (1) changes in Twitter-expressed emotions before and after the first COVID-19 vaccine administration and (2) county-level vaccination rates recorded on March 12 and April 30, 2021—approximately three and four months post-introduction, respectively. March 12 marked the administration of 100 million COVID-19 vaccine doses in the United States, while by April 30, all U.S. states had extended vaccine eligibility to residents aged 16 and older.[1,2] County-level vaccination data were obtained from the U.S. Centres for Disease Control and Prevention (CDC).

Before conducting bivariate correlation, we applied a threshold to filter out extreme values of emotional change, primarily to reduce the influence of outliers that may distort bivariate correlation estimates. This decision was made after inspecting the distribution of emotion change variables and observing a small number of counties (N = 2 out of 1440) with large, potentially spurious shifts, likely due to data sparsity or anomalous activity (e.g., unusually low tweet volume or high variance in small counties). Specifically, we applied thresholds of ±0.15 for sadness and fear variables, ±0.10 for anger, and ±0.25 for joy. We also excluded counties with missing data or zero vaccination rates. This filtering process preserved over 90% of the sample while removing potentially influential outliers, and is consistent with standard practice in large-scale social media analyses where small, normalized changes (e.g., z-scores or relative shifts) are the norm.

After excluding extreme values and zero-dose counties, we found a small but significant positive association between increased expressions of sadness and county-level vaccination rates (For March 12: r = .109, *P* < .001; For April 30: r = 0.08, *P* = .003). This effect was specific to sadness, as other emotions such as fear, anger, and joy showed no significant correlations (See eTables 10 and 11, and eFigure 4 below).

eTable 10. Bivariate Correlation Between Change of Emotions and Vaccination Rates on March 12, 2021

Emotion change	Estimate	t	*p*	*df*	95% CI (Lower)	95% CI (Upper)
Fear	0.019	0.702	0.483	1313	-0.035	0.073
Anger	0.037	1.34	0.181	1313	-0.017	0.091
Joy	-0.005	-0.192	0.848	1313	-0.059	0.049
Sadness	0.109	3.96	< .001	1313	0.055	0.162

Notes. Each row represents a distinct emotion’s change before and after the first COVID-19 vaccine administration; Correlations were conducted independently for each emotion in relation to vaccination rates.

eTable 11. Bivariate Correlation Between Change of Emotions and Vaccination Rates on April 30, 2021

Emotion change	Estimate	t	*P*	*df*	95% CI (Lower)	95% CI (Upper)
Fear	0.009	0.323	0.747	1304	-0.045	0.063
Anger	0.027	0.977	0.329	1304	-0.027	0.081
Joy	0.002	0.08	0.936	1304	-0.052	0.057
Sadness	0.082	2.99	0.003	1304	0.028	0.136

Notes. Each row represents a distinct emotion’s change before and after the first COVID-19 vaccine administration; Correlations were conducted independently for each emotion in relation to vaccination rates.

© 2026 Jaidka K et al. *JAMA Network Open*.

eFigure 4. Bivariate Correlation Between Emotion Changes and Vaccination Rates

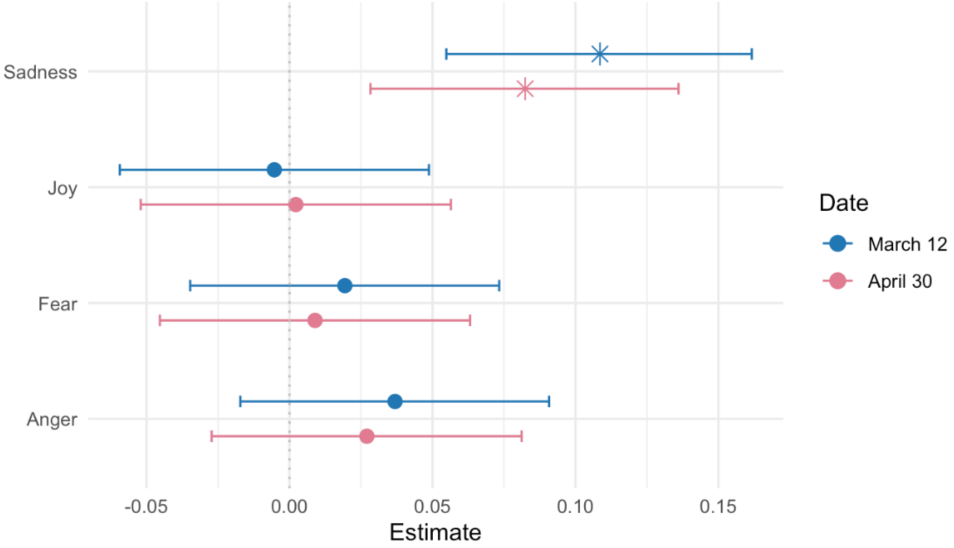

Notes. Blue dots represent correlations with vaccination rates as of March 12, 2021, while red dots represent correlations with vaccination rates as of April 30, 2021. Horizontal whiskers indicate 95% confidence intervals for each correlation coefficient.

Our analysis revealed a small but statistically significant positive association between changes in sadness expression following the first COVID-19 vaccine administration in the U.S. and subsequent vaccination rates three months post-event. However, the causal mechanisms underlying this correlation remain unclear. The observed sadness responses could be attributed to various factors, including emotional reactions to pro-vaccine messaging or personal reflections on pandemic-related losses as vaccines became available. Further qualitative content analysis of tweets would be necessary to disentangle these potential explanations. It is important to acknowledge that our current analysis is limited to bivariate correlations and does not account for potential confounding variables. Vaccination rates are influenced by multiple sociodemographic factors, including age distribution, population density, educational attainment, socioeconomic status, and healthcare access.[3] Future research should employ multivariate analytical approaches, including regression models with appropriate controls, to establish more robust relationships between emotional responses and public health behavior adoption in the context of scientific breakthroughs.

© 2026 Jaidka K et al. *JAMA Network Open*.

eAppendix 10. Placebo Tests at Alternative Event Dates

eTable 12 presents results from placebo interrupted time series models using 8 alternative event dates (e.g., WHO pandemic declaration, state reopenings, election day). The purpose is to assess whether the emotional shifts attributed to vaccine rollout (December 14, 2020) are distinguishable from shifts associated with other major events during the study period. Level-shift coefficients are reported for fear, joy, anger, and sadness at each placebo date. Fear was the emotion most consistent with a vaccine-specific response: the level-shift coefficient reversed sign from positive (pre–December 11) to negative (December 11 onward), indicating that fear was increasing relative to the pre-period trend before the vaccine rollout and decreasing afterward. This sign reversal was unique to fear and was not observed for any other key emotion. The largest fear decrease occurred at the Biden Inauguration ($\beta_2 = -0.834$), suggesting that multiple post-vaccine events contributed to the sustained reduction in fear expression. Joy and anger produced significant positive level shifts at all 8 event dates, with monotonically declining magnitudes from November through January, indicating a gradual downward trend rather than a discrete response to any single event. Sadness was nonsignificant at 6 of 8 dates and significant only at the Capitol Riot and Biden Inauguration.

eTable 12. Placebo Tests With 8 Placebo Events

Event	Date	Emotion	β Interv	β Interact	p Interv	p Interact
Election Day (Nov 3)	3/11/2020	Fear	0.569	-3.100e-05	< .001	< .001
		Anger	0.689	-3.700e-05	< .001	< .001
		Joy	1.097	-5.900e-05	< .001	< .001
		Sadness	0.033	-2.000e-06	0.557	0.556
Election Called (Nov 7)	7/11/2020	Fear	0.724	-3.900e-05	< .001	< .001
		Anger	0.773	-4.200e-05	< .001	< .001
		Joy	1.112	-6.000e-05	< .001	< .001
		Sadness	0.037	-2.000e-06	0.484	0.482
Pfizer EUA Announce (Nov 9)	9/11/2020	Fear	0.665	-3.600e-05	< .001	< .001
		Anger	0.690	-3.700e-05	< .001	< .001
		Joy	1.200	-6.500e-05	< .001	< .001
		Sadness	0.064	-3.000e-06	0.206	0.204
FDA EUA Pfizer (Dec 11)	11/12/2020	Fear	-0.302	1.600e-05	< .001	< .001
		Anger	0.520	-2.800e-05	< .001	< .001
		Joy	0.738	-4.000e-05	< .001	< .001
		Sadness	0.003	0.000e+00	0.923	0.918
First US Dose (Dec 14)	14/12/2020	Fear	-0.424	2.300e-05	< .001	< .001
		Anger	0.445	-2.400e-05	< .001	< .001
		Joy	0.683	-3.700e-05	< .001	< .001

© 2026 Jaidka K et al. *JAMA Network Open*.

		Sadness	-0.004	0.000e+00	0.914	0.921
Moderna EUA (Dec 18)	18/12/2020	Fear	-0.560	3.000e-05	< .001	< .001
		Anger	0.385	-2.100e-05	< .001	< .001
		Joy	0.607	-3.300e-05	< .001	< .001
		Sadness	0.001	0.000e+00	0.975	0.979
Capitol Riot (Jan 6)	6/1/2021	Fear	-0.394	2.100e-05	< .001	< .001
		Anger	0.138	-8.000e-06	< .001	< .001
		Joy	0.463	-2.500e-05	< .001	< .001
		Sadness	0.332	-1.800e-05	< .001	< .001
Biden Inauguration (Jan 20)	20/1/2021	Fear	-0.834	4.500e-05	< .001	< .001
		Anger	-0.028	1.000e-06	0.533	0.552
		Joy	0.216	-1.200e-05	< .001	< .001
		Sadness	0.315	-1.700e-05	< .001	< .001

© 2026 Jaidka K et al. *JAMA Network Open*.

eAppendix11. Sensitivity Analyses With Shifted Intervention Dates

eTable 13 reports results from interrupted time series models in which the intervention date was shifted ±7 and ±14 days from December 14, 2020, to assess sensitivity of findings to the precise specification of the intervention point. Level-shift and slope-change coefficients are reported for all 13 emotions at each alternative date.

The direction of the level-shift coefficient for fear reversed between −14 days (positive) and the primary date (negative), consistent with the vaccine rollout marking a turning point specifically for fear. Joy and anger level shifts were significant and positive at all 5 dates, with magnitudes that increased monotonically as the date shifted earlier — consistent with a gradual trend rather than a sharp break at any single date. Sadness was nonsignificant at 3 of 5 dates. Slope-change coefficients (β_3) were directionally consistent across all shifts for all 4 key emotions.

eTable 13. ±7 and ±14 Day Intervention Shifts

Emotion	Shift	Date	Intervention β (SE)	p	Interaction β (SE)	p
Fear	-14	30/11/2020	0.300 (0.047)	< .001	-1.600e-05 (3.000e-06)	< .001
Anger			0.863 (0.033)	< .001	-4.700e-05 (2.000e-06)	< .001
Joy			1.195 (0.045)	< .001	-6.400e-05 (2.000e-06)	< .001
Sadness			0.093 (0.038)	.016	-5.000e-06 (2.000e-06)	.015
Fear	-7	7/12/2020	-0.108 (0.045)	.015	6.000e-06 (2.000e-06)	.020
Anger			0.648 (0.032)	< .001	-3.500e-05 (2.000e-06)	< .001
Joy			0.841 (0.043)	< .001	-4.500e-05 (2.000e-06)	< .001
Sadness			0.020 (0.037)	.591	-1.000e-06 (2.000e-06)	.583
Fear	0	14/12/2020	-0.424 (0.044)	< .001	2.300e-05 (2.000e-06)	< .001
Anger			0.445 (0.031)	< .001	-2.400e-05 (2.000e-06)	< .001
Joy			0.683 (0.042)	< .001	-3.700e-05 (2.000e-06)	< .001
Sadness			-0.004 (0.036)	.914	0.000e+00 (2.000e-06)	.921
Fear	7	21/12/2020	-0.637 (0.045)	< .001	3.400e-05 (2.000e-06)	< .001
Anger			0.345 (0.032)	< .001	-1.900e-05 (2.000e-06)	< .001
Joy			0.583 (0.043)	< .001	-3.100e-05 (2.000e-06)	< .001
Sadness			0.021 (0.037)	.565	-1.000e-06 (2.000e-06)	.573
Fear	14	28/12/2020	-0.613 (0.047)	< .001	3.300e-05 (3.000e-06)	< .001
Anger			0.261 (0.033)	< .001	-1.400e-05 (2.000e-06)	< .001
Joy			0.496 (0.045)	< .001	-2.700e-05 (2.000e-06)	< .001
Sadness			0.201 (0.038)	< .001	-1.100e-05 (2.000e-06)	< .001

© 2026 Jaidka K et al. *JAMA Network Open*.

eAppendix 12. Robustness Checks: Post-Volume Weighting and County Exclusion

eTables 14 and 15 presents results from two robustness specifications: (1) models weighted by log tweet volume to account for differential county-level posting activity, and (2) models excluding counties with fewer than 30 average daily tweets. Level-shift and slope-change coefficients are reported for all 13 emotions under each specification.

Across both specifications, the direction and significance of level-shift coefficients were consistent with the primary analysis for 12 of 13 emotions. Fear decreased (primary $β_2$ = −0.414, weighted = −0.321, excluded = −0.117; all P < .001), joy increased (primary = 0.097, weighted = 0.714, excluded = 0.917; all P < .001), and anger increased (primary = 0.041, weighted = 0.486, excluded = 0.488; all P < .001). Sadness was the only emotion where results diverged: nonsignificant in the primary ($β_2$ = −0.012, P = .91) and weighted ($β_2$ = −0.033, P = .18) models, but significant after excluding low-volume counties ($β_2$ = 0.054, P = .009). LIWC anger also lost significance in the excluded-county specification ($β_2$ = −0.017, P = .10). Slope-change coefficients ($β_3$) were directionally consistent across all three specifications for all 13 emotions.

eTable 14. Main Effects After Weighting by Post Volume Log Tweet Weighted

Emotion	β Interv	β Interact	p Interv	p Interact
Fear	-0.321	1.700e-05	< .001	< .001
Anger	0.486	-2.600e-05	< .001	< .001
Joy	0.714	-3.800e-05	< .001	< .001
Sadness	-0.033	2.000e-06	0.178	0.183

eTable 15. Main Effects After Excluding Counties With Less Than 30 Posts

Emotion	β Interv	β Interact	p Interv	p Interact
Fear	-0.117	6.000e-06	< .001	< .001
Anger	0.488	-2.600e-05	< .001	< .001
Joy	0.917	-4.900e-05	< .001	< .001
Sadness	0.054	-3.000e-06	0.009	0.008

eReferences

1. Anthes E, Ngo M, Sullivan E. Adults in all U.S. states are now eligible for vaccination, hitting Biden's target. Half have had at least one dose. *The New York Times*. https://www.nytimes.com/2021/04/19/world/adults-eligible-covid-vaccine.html. April 19, 2021. Accessed May 6, 2025.

2. Armour S, Siddiqui S, Ansari T. U.S. Surpasses 100 Million Covid-19 Vaccines Administered. The Wall Street Journal. March 13, 2021. Accessed May 6, 2025. https://www.wsj.com/livecoverage/covid-2021-03-12/card/uXlppHHi6vUu68yTcXOb

3. Cheng X, Mayya R, Sedoc J. To Err Is Human; To Annotate, SILICON? Reducing Measurement Error in LLM Annotation. arXiv preprint arXiv:2412.14461. 2024 Dec 19.

4. Davani AM, Díaz M, Prabhakaran V. Dealing with disagreements: Looking beyond the majority vote in subjective annotations. Transactions of the Association for Computational Linguistics. 2022 Jan 31;10:92-110.

5. Hartmann J. Emotion-english-distilroberta-base. Hugging Face. 2022. Accessed April 1, 2026. https://huggingface.co/distilbert/distilroberta-base

6. Jaidka K. Talking politics: Building and validating data-driven lexica to measure political discussion quality. Computational Communication Research. 2022 Oct;4(2):486-527.

7. Jaidka K, Ahuja H, Ng LH. It takes two to negotiate: Modeling social exchange in online multiplayer games. Proceedings of the ACM on Human-Computer Interaction. 2024 Apr 23;8(CSCW1):1-22.

8. Kreps SE, Kriner DL. Factors influencing Covid-19 vaccine acceptance across subgroups in the United States: Evidence from a conjoint experiment. *Vaccine*. 2021;39(24):3250-3258. doi:10.1016/j.vaccine.2021.04.044

9. Passonneau RJ, Carpenter B. The benefits of a model of annotation. *Transactions of the Association for Computational Linguistics*. 2014;2:311-26.

10. Verma P, Jaidka K, Churina S. "Reasoning" with Rhetoric: On the Style-Evidence Tradeoff in LLM-Generated Counter-Arguments. In Proceedings of the International AAAI Conference on Web and Social Media 2025 Jun 7 (Vol. 19, pp. 1966-1989).

© 2026 Jaidka K et al. *JAMA Network Open*.
