# Supplementary material for: Partisan and Geographic Variation in Emotional Responses to COVID-19 Vaccination on Social Media
Source: JAMA Netw Open. 2026 Jun 1;9(6):e2615409. doi: 10.1001/jamanetworkopen.2026.15409 (PMC13227315; doi:10.1001/jamanetworkopen.2026.15409)
Supplement: Supplement 2. — Data Sharing Statement [file jamanetwopen-e2615409-s002.pdf]

Data Sharing Statement

Jaidka. Partisan and Geographic Variation in Emotional Responses to the COVID-19 Vaccination on Twitter. *JAMA Netw Open*. Published May 28, 2026.
doi:10.1001/jamanetworkopen.2026.15409

Data
Data available: Yes
Data types: Data (not involving human participants)
How to access data: https://osf.io/7xw4v/?view_only=d5b0502f45e04f2fa7abed00d0db783c
When available: With publication

Supporting Documents
Document types: Statistical/analytic code
How to access documents: https://osf.io/7xw4v/?view_only=d5b0502f45e04f2fa7abed00d0db783c
When available: With publication

Additional Information
Who can access the data: anyone requesting the data
Types of analyses: for any purpose
Mechanisms of data availability: with investigator support
